# Dissection of two quantitative trait loci with pleiotropic effects on plant height and spike length linked in coupling phase on the short arm of chromosome 2D of common wheat (*Triticum aestivum* L.)

**DOI:** 10.1007/s00122-019-03318-z

**Published:** 2019-03-26

**Authors:** Lingling Chai, Zhaoyan Chen, Ruolin Bian, Huijie Zhai, Xuejiao Cheng, Huiru Peng, Yingyin Yao, Zhaorong Hu, Mingming Xin, Weilong Guo, Qixin Sun, Aiju Zhao, Zhongfu Ni

**Affiliations:** 1 State Key Laboratory for Agrobiotechnology/Key Laboratory of Crop Heterosis and Utilization, the Ministry of Education/Key Laboratory of Crop Genetic Improvement, Beijing Municipality, China Agricultural University, Beijing, 100193 China; 2 National Plant Gene Research Centre, Beijing, 100193 China; 3 College of Agronomy, Henan Agricultural University, Zhengzhou, 450002 China; 4 Institute of Cereal and Oil Crops, Hebei Academy of Agriculture/Forestry Sciences, Hebei Crop Genetic Breeding Laboratory, Shijiazhuang, 050035 China

## Abstract

**Key message:**

Two QTL with pleiotropic effects on plant height and spike length linked in coupling phase on chromosome 2DS were dissected, and diagnostic marker for each QTL was developed.

**Abstract:**

Plant height (PHT) is a crucial trait related to plant architecture and yield potential, and dissection of its underlying genetic basis would help to improve the efficiency of designed breeding in wheat. Here, two quantitative trait loci (QTL) linked in coupling phase on the short arm of chromosome 2D with pleiotropic effects on PHT and spike length, *QPht/Sl.cau*-*2D.1* and *QPht/Sl.cau*-*2D.2,* were separated and characterized. *QPht/Sl.cau*-*2D.1* is a novel QTL located between SNP makers *BS00022234_51* and *BobWhite_rep_c63957_1472. QPht/Sl.cau*-*2D.2* is mapped between two SSR markers, *SSR*-*2062* and *Xgwm484*, which are located on the same genomic interval as *Rht8*. Moreover, the diagnostic marker tightly linked with each QTL was developed for the haplotype analysis using diverse panels of wheat accessions. The frequency of the height-reduced allele of *QPht/Sl.cau*-*2D.1* is much lower than that of *QPht/Sl.cau*-*2D.2*, suggesting that this novel QTL may be an attractive target for genetic improvement. Consistent with a previous study of *Rht8*, a significant difference in cell length was observed between the NILs of *QPht/Sl.cau*-*2D.2*. By contrast, there was no difference in cell length between NILs of *QPht/Sl.cau*-*2D.1*, indicating that the underlying molecular mechanism for these two QTL may be different. Collectively, these data provide a new example of QTL dissection, and the developed diagnostic markers will be useful in marker-assisted pyramiding of *QPht/Sl.cau*-*2D.1* and/or *QPht/Sl.cau*-*2D.2* with the other genes in wheat breeding.

**Electronic supplementary material:**

The online version of this article (10.1007/s00122-019-03318-z) contains supplementary material, which is available to authorized users.

## Introduction

Wheat (*Triticum aestivum* L.) is an important food crop worldwide, providing calories and proteins consumed by humankind (Fischer et al. 2014; Shiferaw et al. 2013). Plant height (PHT) is a crucial trait related to plant architecture and yield potential (Cadalen et al. 1998; Peng et al. 1999; Sakamoto and Matsuoka 2004). Consequently, the use of dwarfing genes to reduce PHT and improve yield has been one of the main strategies in breeding modern high-yielding hexaploid bread wheat varieties. For example, during the green revolution, the introduction of semi-dwarf varieties into wheat (*T. aestivum* L.) appropriately reduced PHT and contributed significantly to a worldwide increase in potential grain yield (Peng et al. 1999). PHT is known to be typically under polygenic control (Bellucci et al. 2015; Tang et al. 2007). Thus, identification of QTL/gene controlling PHT would help to improve the efficiency of designed breeding in wheat.

To date, 24 genes influencing PHT have been identified and designated reduced height genes in wheat (McIntosh et al. 2017). Of these 24 major genes, *Rht*-*B1b* and *Rht*-*D1b*, which are located on chromosomes 4B and 4D, respectively (Pearce et al. 2011), were cloned and most widely used in wheat breeding (Borner et al. 2002; Cadalen et al. 1998). They encoded DELLA proteins, which are transcriptional regulators that reduce response to gibberellin. Thus, *Rht*-*B1b* and *Rht*-*D1b* belonged to the group of dwarfing genes that is insensitive to gibberellic acid (Pearce et al. 2011). The characterization of these two genes has enhanced our knowledge about PHT determination in wheat, and functional markers developed for them have been used in wheat breeding (Akman and Bruckner 2012; Borrell et al. 1991; Tang et al. 2009).

The other extensively used gene in wheat breeding is *Rht8*, which is derived from the Japanese variety Akakomugi and was introduced into Southern European wheat breeding in the 1930s by the Italian breeder Strampelli, together with the photoperiod-insensitive, early flowering *Ppd*-*D1a* allele (Lorenzetti 2000). *Rht8* was mapped on the short arm of chromosome 2D, and a closely linked SSR marker named *Xgwm261* was detected (Korzun et al. 1998). The 192-bp allele of *Xgwm261* corresponds to a height-reducing phenotype of *Rht8*, attributing to a 7–8 cm reduction in PHT without pleiotropic effects on other agronomic traits (Korzun et al. 1998; Worland et al. 1998). Since then, the ‘diagnostic’ 192-bp allele was used to survey the presence of *Rht8* in wheat cultivars (Ahmad and Sorrells 2002; Asplund et al. 2012; Bai et al. 2004; Chebotar et al. 2001; Liu et al. 2005; Worland et al. 2001a, b; Zhang et al. 2006). However, the 192-bp allele of *Xgwm261* was not always linked to *Rht8* (Ellis et al. 2007). A recent study showed that *Rht8* is located in a genetic interval of 1.29 cM (*DG279*-*DG371*), which is 1.95 cM away from *Xgwm261* (Gasperini et al. 2012). Thus, the development of a robust marker tightly linked to *Rht8* is the priority for marker-assisted selection.

Over the past two decades, the successful application of quantitative–genetic methodology has facilitated identification of numerous QTL for PHT in wheat (Borner et al. 2002; Cadalen et al. 1998; Wurschum et al. 2015, 2017; Yu et al. 2014; Zanke et al. 2014). In our recent study, two QTL (*QPht.cau*-*2D.1* and *QPht.cau*-*2D.2*) for PHT linked in coupling phase were mapped on the short arm of chromosome 2D (Zhai et al. 2016). Since both QTL are located on chromosome 2DS, this investigation was undertaken to determine the genetic relationship between *QPht.cau*-*2D.1* and *QPht.cau*-*2D.2* using segregating populations and NILs derived from a residual heterozygous line. Moreover, the diagnostic marker for each QTL was developed for marker-assisted selection in wheat breeding programmes. In addition, haplotype distribution of *QPht.cau*-*2D.1* and *QPht.cau*-*2D.2* in diverse panels of wheat accessions was also investigated.

## Materials and methods

### Plant materials

**Fig. 1:**
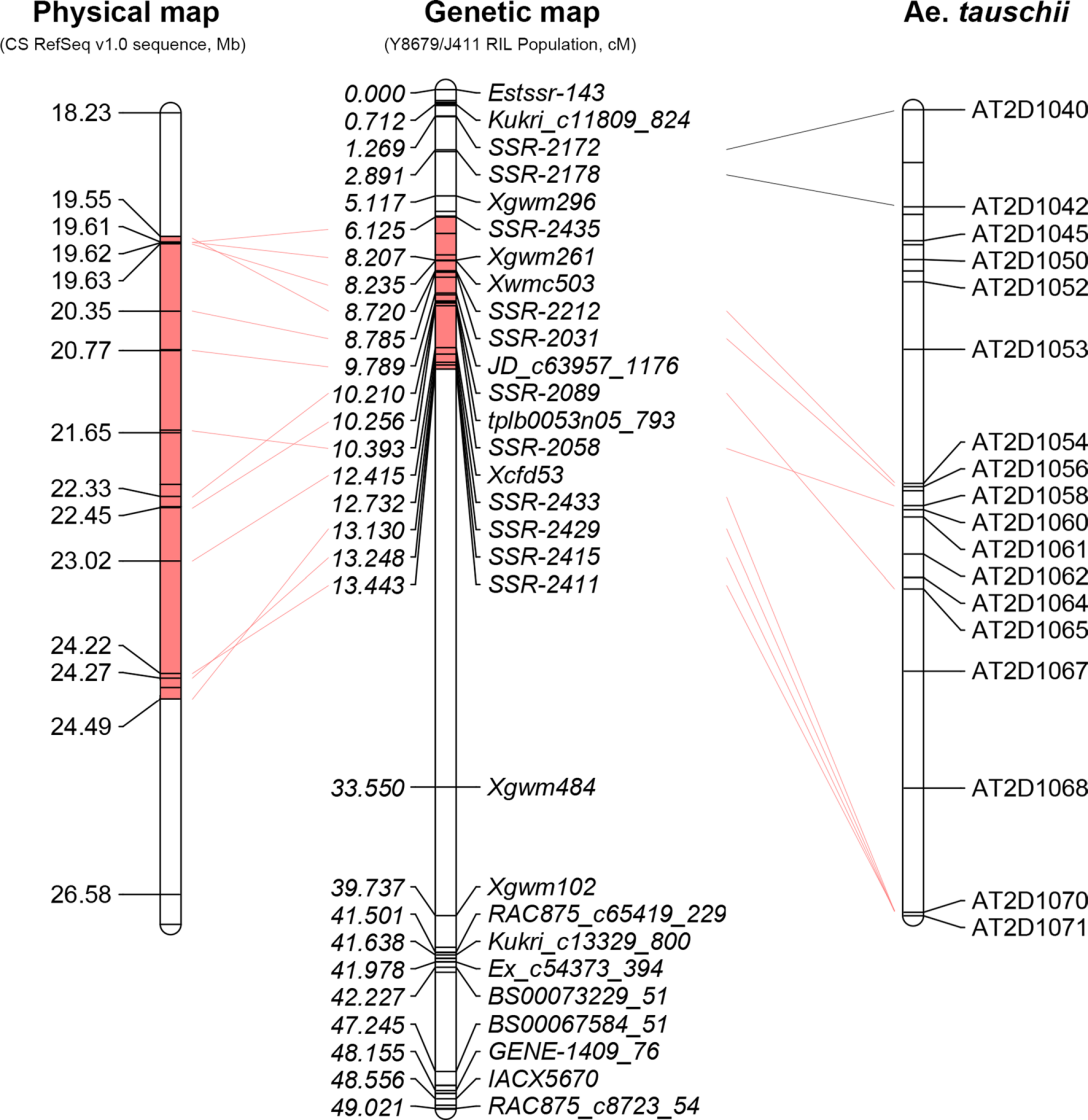
Saturated genetic linkage map of chromosome 2D in the RIL population and the collinearity of the developed markers, *Aegilops tauschii* markers and corresponding physical position in the Chinese Spring RefSeq v1.0 sequence. The red segment means the heterozygous segment in RIL171 (color figure online)

**Fig. 2:**
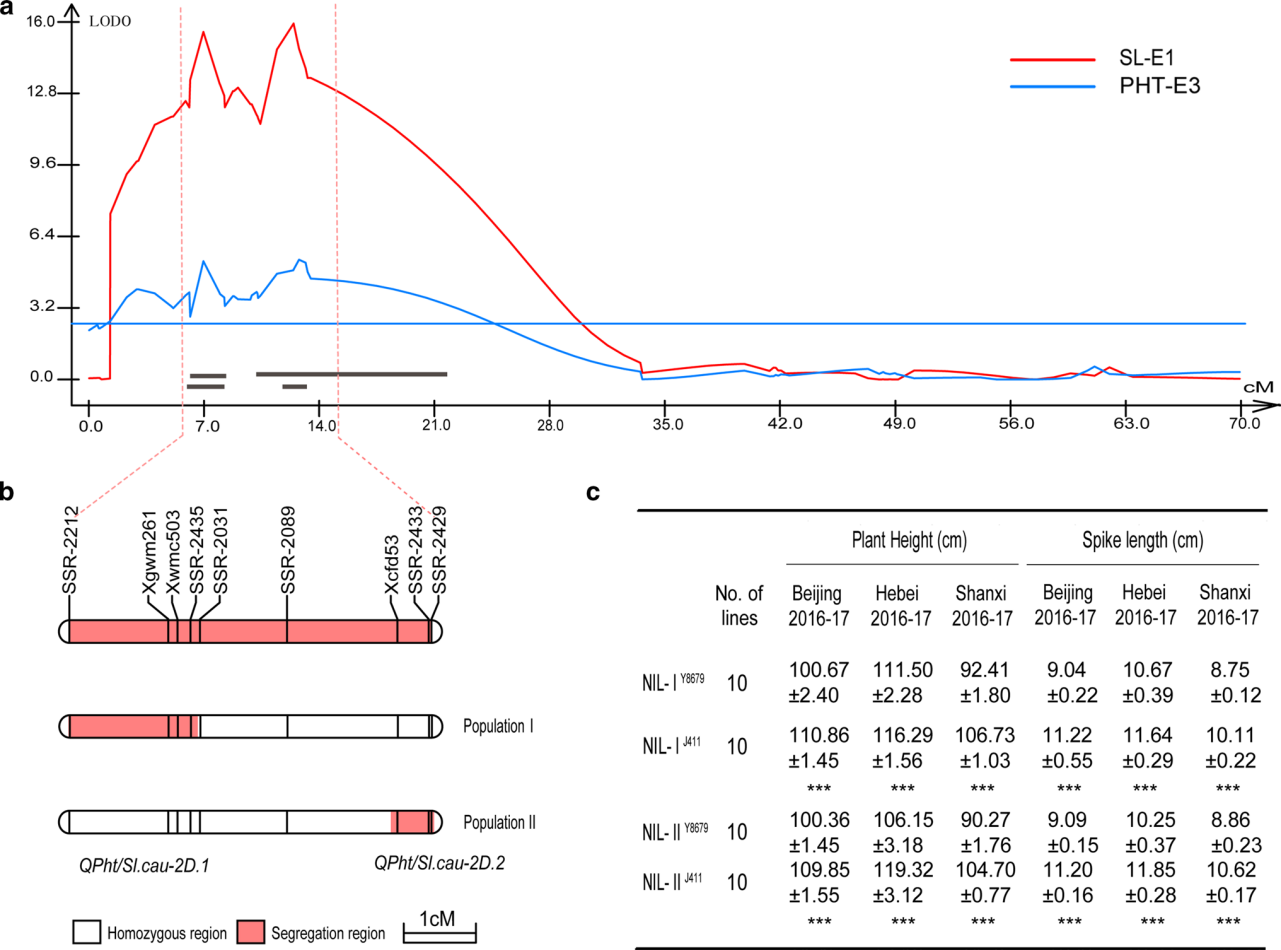
Dissection of *QPht/Sl.cau*-*2D.1* and *QPht/Sl.cau*-*2D.2*. **a** QTL mapping using a saturate genetic map. **b** Graphical genotypes of two populations (derived from RIL 171). **c** Performance of the members of two NIL pairs in three field trails. *** indicate significant differences at the 0.001 levels (Student’s *t* test)

Following our previous study of QTL mapping using the recombinant inbred lines (RILs) of Yumai 8679 (Y8679)/Jing 411 (J411) (Zhai et al. 2016), SSR (simple sequence repeat) markers were further developed for linkage map construction and QTL analysis. In generation F_9_ of the RIL population, a RHL (RIL171) that carried the heterozygous segment at the genetic region from SSR markers *SSR*-*2212* to *SSR*-*2429* was self-pollinated to produce the F_10_ generation for further study (Fig. 1). The homozygotes (F_10_) without recombinant were selected as NILs (NIL^Y8679^ and NIL^J411)^ to validate the presence of the QTL. Two plants that carried heterozygous segments covering the intervals from *SSR*-*2212* to *Xwmc503* and from *Xcfd53* to *SSR*-*2429* were identified to produce segregate populations (F_11_, population I and II) for genetic analysis (Fig. 2b). Then, the non-recombinant homozygotes of each population were selected and self-pollinated to produce NIL-I (F_12_, NIL-I^Y8679^ and NIL-I^J411^) and NIL-II (F_12_, NIL-II^Y8679^ and NIL-II^J411^).

In total, 1433 wheat accessions with varying ploidy were used to test the allele frequency of two diagnostic markers (*STARP*-*2001* and *SSR*-*2433*). These included 1347 hexaploid wheat accessions (724 Chinese varieties/lines, 181 Chinese mini core accessions and 442 accessions from other countries (Supplementary Tables S1, S2 and S3, respectively), and 86 diploid accessions (*Aegilops tauschii*; Supplementary Table S4).

### Field experiments

Populations I and II were tested in Hebei (37°56′N, 114°42′E). NIL^Y8679^ and NIL^J411^ were grown in two locations, Shanxi (36°08′N, 111°34′E) and Hebei, and NIL-I and NIL-II were grown in three locations, Beijing, Shanxi and Hebei (Supplementary Table S5). In each environment, two segregating populations were planted with a sowing rate of 20 seeds per row (1.5 m long and 0.3 m apart), while the NILs were planted 25 seeds per line. The irrigation and other management of all sites were in accordance with local standard practices.

### Phenotypic evaluation and statistical analysis

Plant height (PHT), spike length (SL) and spikelet number (SN) of the individuals in populations I and II were measured before harvest. For the NILs, five plants from each line were used for phenotypic evaluation. PHT, SL, SN and length of 5 internodes from the main tillers were measured. For NILs, the mean values over three replications at each site were used for data analysis. Spikelet compactness (SC) was calculated by dividing the SN by the SL.

Statistical analysis was performed with IBM SPSS Statistics 20 (SPSS, Chicago, USA). Significance analysis was calculated using Student’s *t* test.

### SSR marker development

Microsatellite markers were designed to construct the genetic map of chromosome 2DS. The reference sequence of wheat variety Chinese Spring (http://www.wheatgenome.org/) and *Aegilops. tauschii* genomic DNA contig sequence (http://aegilops.wheat.ucdavis.edu/ATGSP/) were extracted to design new SSR markers by using BLASTN method with the sequences of markers *DG279* and *DG371* (Gasperini et al. 2012) and the flanking sequence of the polymorphic SNPs in our previous results (Zhai et al. 2016). The PCR system included 5 μl 2 × Taq PCR StarMix, 2 μl primer (mixture of left and right primer), 2 μl DNA template (50–100 ng/μl) and 1 μl H_2_O. The PCR protocol was performed using PCR amplification as follows: 94 °C for 5 min; 36 cycles of 94 °C denaturation for 30 s, 55–57 °C primer annealing for 30 s, and 72 °C extending for 30 s; and finally, 72 °C for 10 min. For polymorphism detection, PCR products were separated using the method of 8% non-denaturing polyacrylamide gel electrophoresis (PAGE) (Marklund et al. 1995). The primers of 17 codominant SSR markers are listed in Supplementary Table S14.

### Linkage map construction and QTL analysis

JoinMap 4.1 was used for constructing genetic linkage map of chromosome 2DS with LOD value above 5. A regression mapping algorithm (Stam 1993) and Kosambi’s mapping function (Kosambi 1943) were applied to determine the marker order and convert recombination into distances.

QTL mapping was determined with Windows QTL Cartographer 2.5 (Wang et al. 2006). Briefly, composite interval mapping (CIM) was performed using model 6 with forward and backward regression, five markers, and a 1-cM scanning window as cofactors. Empirical threshold LOD scores estimated with 1000 permutations at *P* ≤ 0.05 detected QTL with overlapping confidence intervals (± 2 LOD away from the peaks of likelihood ratios) were considered equivalent and named as suggested by McIntosh et al. (2017).

### STARP marker development

To develop a diagnostic marker for *QPht/Sl.cau*-*2D.1*, the SNP marker *AX*-*108988107* was converted into a semi-thermal asymmetric reverse marker (STARP) (Long et al. 2017). The primers included two asymmetrically modified AMAS primers (STARP-2001 F1 and STARP-2001 F2) and their same reverse primer (STARP-2001 R). STARP-2001 F1 was designed to amplify the Y8679 allele uniquely with 10 bp insertion (TGCTGACGAC) at 5′ terminus. Meanwhile, STARP-2001 F2 could amplify the J411 allele. The nucleotides substitution principle followed the suggestion of Long et al. (Long et al. 2017). The three primers were mixed in a ratio of 1:1:2 (STARP-2001 F1: STARP-2001 F2: STARP-2001 R) and diluted. A 10-μl PCR system containing 5 μl 2 × Taq PCR StarMix, 2 μl mixed primer, 2 μl DNA template (50–100 ng/μl) and 1 μl H_2_O was performed with 94 °C initial denaturation for 5 min, followed by 11 cycles of a 3-step touchdown PCR protocol starting at 94 °C for 30 s, then 66 °C for 30 s and 72 °C extending for 30 s, with the annealing temperature being decreased by 1 °C per cycle. This touchdown PCR protocol was accompanied with 30 cycles of 3 steps (94 °C for 30 s, then 55 °C for 30 s and 72 °C extending for 30 s) and then, finally, 72 °C for 10 min. Then, 8% PAGE was adopted to separate the length polymorphism.

### Histological analysis

The medial sections of peduncle at flowering stage were collected from NIL-I^Y8679^, NIL-I^J411,^ NIL-II^Y8679^ and NIL-II^J411^ plants, then fixed in FAA solution (50% (v/v) ethanol, 5% (v/v) glacial acetic acid and 4% (v/v) formaldehyde) and subjected to vacuum pumping for 30 min. Next, the internodes were dehydrated in a series of ethanol solutions (75% (v/v) ethanol, 85% (v/v) ethanol, 90% (v/v) ethanol, 95% (v/v) ethanol and anhydrous ethanol) and destained in a series of xylene solutions (3:1 ethanol: xylene, 1:1 ethanol: xylene, 1:3 ethanol: xylene, and pure xylene). The internodes were soaked in each ethanol and xylene solution for 1 h and then embedded in paraffin (He et al. 2017). Tissue sections were cut into 4-μm-thick slices, fixed on a glass slide, and stained with 1% sarranine and 0.5% fast green (G1031, http://www.servicebio.cn/).

### Sequence analysis of candidate genes

Genomic DNA for Y8679 and J411 was used to build paired-end sequencing libraries with insert sizes of approximately 500 bp, according to vendor-provided instructions (Illumina). An average 5 × coverage of the assembled genome, with 150-bp paired-end reads for each accession, was generated with the Illumina HiSeq X Ten platform. All the sequence reads for each parent were mapped to the newly updated genome of Chinese Spring (RefSeq v1.0) in the Burrows–Wheeler Aligner program (BWA, ver. 0.7.15) with default parameters (Li and Durbin 2009). SNPs and InDels are identified by the HaplotypeCaller module. The sequence of each candidate gene including intron, exon, 3′-UTR region and 2-kb sequence upstream of translation start codon was used as query for analysis.

## Results

### Saturation of genetic Linkage map and QTL mapping of *QPht/Sl.cau*-*2D.1* and *QPht/Sl.cau*-*2D.2*

To saturate the genetic linkage map for plant height (PHT) and spike length (SL) on the short arm of chromosome 2D, 632 SSR markers were developed, among which 17 markers exhibited polymorphism between parents Y8679 and J411 (Supplementary Table S14). The collinearity of these markers with *Ae. tauschii* markers was analysed, and the physical positions on the reference sequence of Chinese Spring are shown in Fig. 1. These 17 polymorphic SSR markers were used to genotype the 191 RIL lines for QTL analysis. The resulting genetic linkage map of chromosome 2D consisted of 75 markers spanning 70.11 cM in length (Supplementary Table S13). Consistent with our previous finding, two stable QTL in coupling phase controlling PHT (*QPht.cau*-*2D.1* and *QPht.cau*-*2D.2*) were detected on chromosome 2DS by reanalysing the phenotypic data from 191 RIL lines in 9 individual environments and 1 combined analysis (BLUP) (Zhai et al. 2016). *QPht.cau*-*2D.1* was located between SNP markers *BS00022234_51* and *BobWhite_rep_c63957_1472*, which explained 4.39–11.94% of the phenotypic variation. *QPht.cau*-*2D.2* was mapped to the genomic interval between SSR markers *SSR*-*2062* and *Xgwm484,* which explained 4.12–12.96% of the phenotypic variations under different environments (Fig. 2a, Table 1). In the same positions of QTL for PHT, two major QTL for SL (*QSl.cau*-*2D.1* and *QSl.cau*-*2D.2*) were also detected under all environments, which explained 30.94–40.63 and 31.31–41.95% of the phenotypic variations, respectively. All increasing alleles for PHT and SL came from J411 (Table 1). For simplification, hereafter, the two genomic regions covering PHT and SL were designated as *QPht/Sl.cau*-*2D.1* and *QPht/Sl.cau*-*2D.2,* respectively.

**Table 1:** Effects of QTL for PHT and SL in individual environments

Trait	QTL	En.	Nearest marker	Position (cM)	Confidence interval		LOD	Add	*R*^2^ (%)
					Left	Right			
SL									
	*QSl.cau*-*2D.1*	E1	*D_GDRF1KQ01AX0PH_169*	6.9	6.1	8.1	15.56	− 0.596	31.37
		E2	*D_GDRF1KQ01AX0PH_169*	6.9	6.6	7.5	16.70	− 0.597	31.38
		E3	*D_GDRF1KQ01AX0PH_169*	6.9	6.4	7.4	15.75	− 0.612	30.94
		E4	*D_GDRF1KQ01AX0PH_169*	6.9	6.6	7.5	17.04	− 0.661	31.74
		E5	*D_GDRF1KQ01AX0PH_169*	6.9	6.6	7.4	17.83	− 0.652	34.81
		E6	*D_GDRF1KQ01AX0PH_169*	6.9	6.6	7.6	16.27	− 0.601	31.16
		E7	*D_GDRF1KQ01AX0PH_169*	6.9	6.5	7.3	18.13	− 0.655	33.19
		E8	*D_GDRF1KQ01AX0PH_169*	6.9	6.1	7.5	21.90	− 0.698	40.63
		E9	*D_GDRF1KQ01AX0PH_169*	6.9	6.7	7.3	20.64	− 0.674	38.33
		E10	*D_GDRF1KQ01AX0PH_169*	6.9	6.6	7.3	21.31	− 0.647	39.16
	*QSl.cau*-*2D.2*	E1	*Xcfd53*	12.4	11.9	13.1	15.94	− 0.599	31.91
		E2	*SSR*-*2433*	12.7	11.2	15.2	18.13	− 0.617	33.49
		E3	*SSR*-*2433*	12.7	12.4	13.2	16.61	− 0.623	32.31
		E4	*Xcfd53*	12.4	11.5	13.8	16.73	− 0.652	31.31
		E5	*SSR*-*2433*	12.7	11.3	13.2	17.26	− 0.641	33.90
		E6	*SSR*-*2433*	12.7	12.6	29.1	18.44	− 0.633	34.45
		E7	*SSR*-*2433*	12.7	12.3	32.5	18.96	− 0.668	34.43
		E8	*SSR*-*2433*	12.7	12.4	33.0	22.88	− 0.707	41.95
		E9	*SSR*-*2433*	12.7	12.4	13.2	21.07	− 0.677	38.89
		E10	*SSR*-*2433*	12.7	12.5	32.3	21.71	− 0.649	39.73
PHT									
	*QPht.cau*-*2D.1*	E1	*D_GDRF1KQ01AX0PH_169*	6.9	6.9	6.9	1.95	− 1.742	4.39
		E3	*D_GDRF1KQ01AX0PH_169*	6.9	6.3	8.2	5.30	− 2.899	11.94
		E5	*D_GDRF1KQ01AX0PH_169*	6.9	6.1	8.2	2.90	− 2.609	6.32
		E7	*D_GDRF1KQ01AX0PH_169*	6.9	6.1	8.0	2.72	− 3.207	6.31
		E8	*D_GDRF1KQ01AX0PH_169*	6.9	6.1	9.0	2.98	− 2.061	6.52
		E10	*D_GDRF1KQ01AX0PH_169*	6.9	6.1	9.0	2.18	− 2.080	4.75
	*QPht.cau*-*2D.2*	E1	*SSR*-*2411*	18.4	13.3	33.5	2.76	− 2.476	9.63
		E2	*Xcfd53*	12.4	12.4	12.4	1.91	− 2.244	4.12
		E3	*SSR*-*2433*	12.7	10.3	21.6	5.37	− 2.904	12.07
		E5	*SSR*-*2433*	12.7	10.3	26.4	3.14	− 2.698	6.80
		E6	*SSR*-*2433*	12.7	10.3	13.3	2.34	− 2.461	5.15
		E7	*Xcfd53*	12.4	10.4	12.4	2.61	− 3.418	7.34
		E8	*SSR*-*2411*	17.4	13.3	28.2	3.83	− 2.790	12.96
		E10	*SSR*-*2433*	12.7	10.3	13.3	2.64	− 2.269	5.72

LOD, maximum likelihood LOD score for the QTLs; Add, ± additive effect. Positive value indicates a positive effect of Y8679, whereas negative value indicates a positive effect of J411; *R*^*2*^(%), phenotype contribution rate; SL, spike length; PHT, plant height; Position, the genetic position of nearest marker to LOD peak; En, the environments of data collection Zhai et al. (2016)

### Development of near isogenic lines harbouring *QPht/Sl.cau*-*2D.1* and *QPht/Sl.cau*-*2D.2*

To further evaluate the genetic effects of *QPht/Sl.cau*-*2D.1* and *QPht/Sl.cau*-*2D.2* on PHT and SL, one line (RIL171) from the F_9_ generation of the RIL population was selected for investigation because of its residual heterozygosity at the target QTL genomic regions. First, one pair of NILs (NIL^Y8679^ and NIL^J411^) from the self-pollinated progeny of RIL171 (F_10_) harbouring *QPht/Sl.cau*-*2D.1* and *QPht/Sl.cau*-*2D.2* were genotyped using the wheat 660 K SNP array (https://wheat.pw.usda.gov/ggpages/topics/Wheat660_SNP_array_developed_by_CAAS.pdf). Of 630517 SNP markers with detected signal, only 434 (0.07%) exhibited polymorphism between NIL^Y8679^ and NIL^J411^, indicating that the genetic backgrounds of these two NILs had very high similarity (Supplementary Table S6). Notably, based on the information of SNP markers with genomic position, 154 of 434 polymorphic SNP markers (35.48%) were located on genomic interval flanking *QPht/Sl.cau*-*2D.1* and *QPht/Sl.cau*-*2D.2* (Supplementary Table S7). Second, the progenies of the above NILs (F_11_) were used for phenotypic analysis. As shown in Table 2, significant differences were observed for SL, length of peduncle (PED) and length of 1st internode under peduncle (1IL) between NIL^Y8679^ and NIL^J411^ (*P *< 0.001) under different environments. By contrast, the differences in length of the second internode under peduncle (2IL), length of the third internode under peduncle (3IL) and length of the fourth internode under peduncle (4IL) were only detected under a specific environment. In addition, no difference was observed for spikelet number (SN). Consequently, PHT of NIL^J411^ was 12.62 and 15.08% (*P *< 0.001) higher than that of NIL^Y8679^ under two different environments. Correspondingly, spikelet compactness (SC) of NIL^Y8679^ was significantly (*P *< 0.001) higher than that of NIL^J411^ (Table 2).

**Table 2:** Effect analysis for different traits in NIL^Y8679^ and NIL^J411^

Trait	Shanxi (Oct. 2015–Jun. 2016)				Hebei (Oct. 2015–Jun. 2016)			
	Phenotypic mean		Deta (%)	*P* value	Phenotypic mean		Deta (%)	*P* value
	NIL^Y8679^	NIL^J411^			NIL^Y8679^	NIL^J411^		
PHT	73.56 ± 1.73	82.84 ± 2.43	12.62	0.000	93.59 ± 3.00	107.70 ± 2.16	15.08	0.000
SL	8.60 ± 0.19	10.29 ± 0.44	19.65	0.000	9.72 ± 0.37	11.41 ± 0.34	17.39	0.000
SN	16.96 ± 0.35	16.84 ± 0.28	− 0.71	0.413	18.27 ± 0.28	18.41 ± 0.29	7.66	0.461
PED	27.28 ± 0.80	33.57 ± 2.27	23.06	0.000	31.03 ± 0.83	36.32 ± 0.88	17.05	0.000
SC	1.98 ± 0.03	1.65 ± 0.07	− 0.33	0.000	2.01 ± 0.09	1.66 ± 0.13	− 0.35	0.000
1IL	20.02 ± 0.88	22.23 ± 1.03	11.04	0.000	22.59 ± 0.65	24.53 ± 0.27	8.59	0.000
2IL	13.19 ± 0.51	13.62 ± 0.74	3.26	0.148	15.13 ± 0.82	16.61 ± 0.82	9.78	0.001
3IL	6.93 ± 0.51	7.13 ± 1.00	2.89	0.587	11.50 ± 0.81	12.17 ± 0.97	5.83	0.107
4IL	2.93 ± 0.74	2.56 ± 0.61	− 12.63	0.238	4.70 ± 0.90	6.74 ± 0.88	43.40	0.000

PHT, the traits are plant height; SL, spike length; PED, the length of peduncle; SC, spikelet compactness; 1IL, length of the first internode under peduncle; 2IL, length of the second internode under peduncle; 3IL, length of the third internode under peduncle; 4IL, length of the fourth internode under peduncle; SN, spikelet number per spike

### Dissection of the two QTL linked in coupling phase

**Fig. 3:**
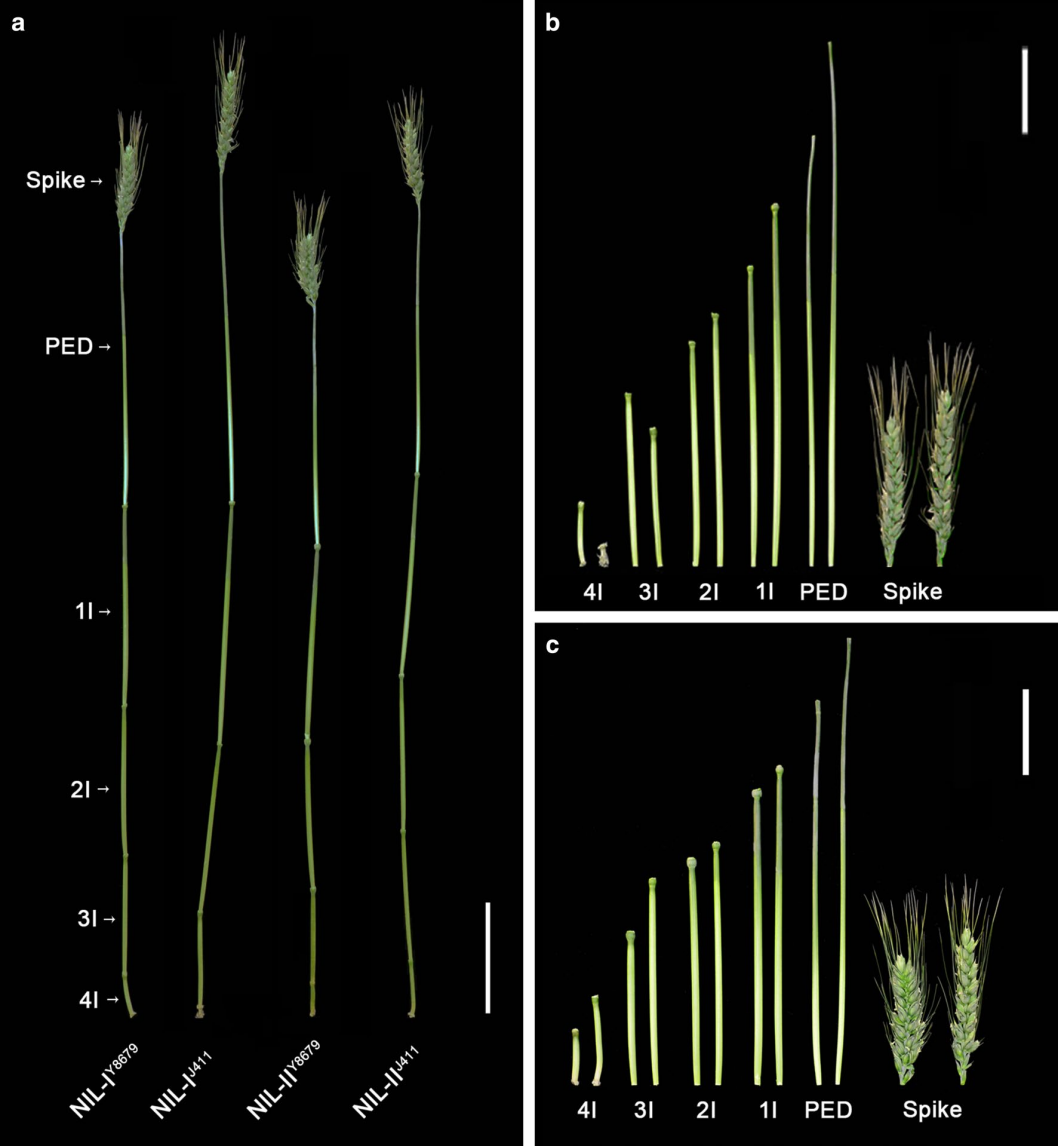
Culm and spike morphology of the NILs of *QPht/Sl.cau*-*2D.1* and *QPht/Sl.cau*-*2D.2* grown in Beijing (2016–2017 growing season). **a** Main tillers. Bars = 10 cm. **b** Spikes, peduncles and other internodes of NIL-I^Y8679^ (left) and NIL-I^J411^ (right). The bar represents 5 cm. **c** Spikes, peduncles and the internodes of NIL-II^Y8679^ (left) and NIL-II^J411^ (right). The bar represents 5 cm. PED, peduncle; 1I, first internode under peduncle; 2I, the second internode under peduncle; 3I, the third internode under peduncle; and 4I, the fourth internode under peduncle

To further investigate the genetic effect of each QTL (*QPht/Sl.cau*-*2D.1* and *QPht/Sl.cau*-*2D.2*), eight polymorphic SSR markers between NIL^J411^ and NIL^Y8679^ were used to genotype the segregating populations of RIL 171 (F_10_). Two plants carrying different heterozygous genomic regions (I and II) were selected for further analysis, corresponding to *QPht/Sl.cau*-*2D.1* and *QPht/Sl.cau*-*2D.2*, respectively (Fig. 2b). The single marker analysis for PHT, SL and SN was performed using the derived segregating populations I and II (F_11_). As expected, polymorphic SSR markers in each population were significantly (*P *< 0.001) associated with PHT and SL (Table 3). To further test the pleiotropic effects of *QPht/Sl.cau*-*2D.1* and *QPht/Sl.cau*-*2D.2*, two sets of NILs (F_12_, NIL-I and NIL-II) (Fig. 3) were developed from the two F_11_ populations. For PHT, SL and PED, NIL-I^J411^ and NIL-II^J411^ (genotype same with J411) had significantly higher values than those of NIL-I^Y8679^ and NIL-II^Y8679^ (genotype same with Y8679) under all environments, with average differences of 9.97% and 12.62, 16.25 and 19.56%, and 13.43% and 18.11%, respectively (Fig. 2c, Supplementary Table S9). In addition, the 1IL and 4IL of NIL-II^J411^ were also much higher (*P *< 0.001) than those of NIL-II^Y8679^ under all environments (Supplementary Table S9).

**Table 3:** Single marker analysis of markers on chromosomes 2D with plant height (PHT), spike length (SL) and spikelet number (SN) in two segregation populations

Population	Marker	PHT			SL			SN		
		*F*(_1,n−2_)	Pr(*F*)	*R* ^2^	*F*(_1,n−2_)	Pr(*F*)	*R* ^2^	*F*(_1,n−2_)	Pr(*F*)	*R* ^2^
Population I	*SSR*-*2212*	20.594	0.000011666****	0.1029	12.741	0.000482821***	0.0655	0.610	0.436151787	0.0086
	*Xwmc503*	19.909	0.000015982****	0.1186	12.289	0.000603258***	0.0767	0.790	0.375456135	0.0053
	*Xgwm261*	20.765	0.000010785****	0.1230	12.888	0.000449152***	0.0801	0.848	0.358628765	0.0057
	*SSR*-*2435*	20.765	0.000010785****	0.1230	12.888	0.000449152***	0.0801	0.848	0.358628765	0.0057
Population II	*Xcfd53*	34.498	0.000000083****	0.2911	18.119	0.000053755****	0.1774	0.092	0.76258363	0.0011
	*SSR*-*2433*	42.318	0.000000005****	0.3350	23.332	0.000006058****	0.2174	0.701	0.404710321	0.0083
	*SSR*-*2429*	42.318	0.000000005****	0.3189	23.333	0.000006056****	0.1891	0.701	0.404689005	0.0049

*** and **** indicate significance at the 0.001 and 0.0001 levels, respectively

**Fig. 4:**
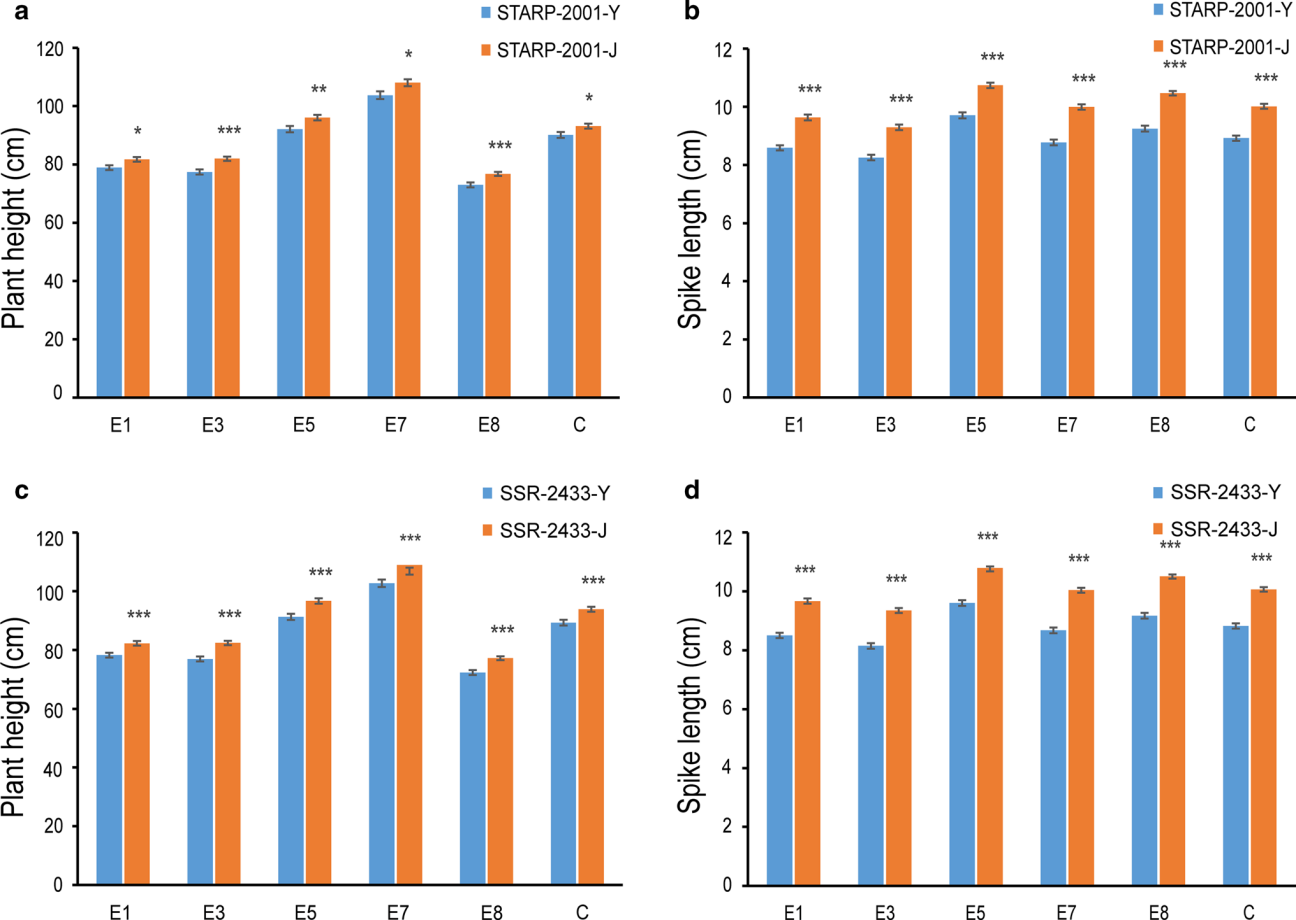
Difference in PHT and SL between two alleles of *STARP*-*2001* and *SSR*-*2433* in the RIL population of five environments and one combined analysis (BLUP). The values represent the means (± SD) of RILs with the same genotype. *, **, *** indicate significant differences at the 0.05, 0.01, 0.001 levels (Student’s *t* test), respectively. STARP-2001-Y, the group with Y8679 type; STARP-2001-J, the group with J411 type; SSR-2433-Y, the group with Y8679 type; SSR-2433-J, the group with J411 type. The *x*-axis, five environments and one combined analysis(BLUP): E1, Beijing, 2010–2011; E3, Beijing, 2011–2012; E5, Anhui, 2012–2013; E7, Shaanxi, 2012–2013; E8, Beijing, 2014–2015; C indicates the combined QTL analysis based on the BLUP values across nine environments. **a** PHT between two alleles of *STARP*-*2001*. **b** SL between two alleles of *STARP*-*2001*. **c** PHT between two alleles of *SSR*-*2433*. **d** SL between two alleles of *SSR*-*2433*

**Fig. 5:**
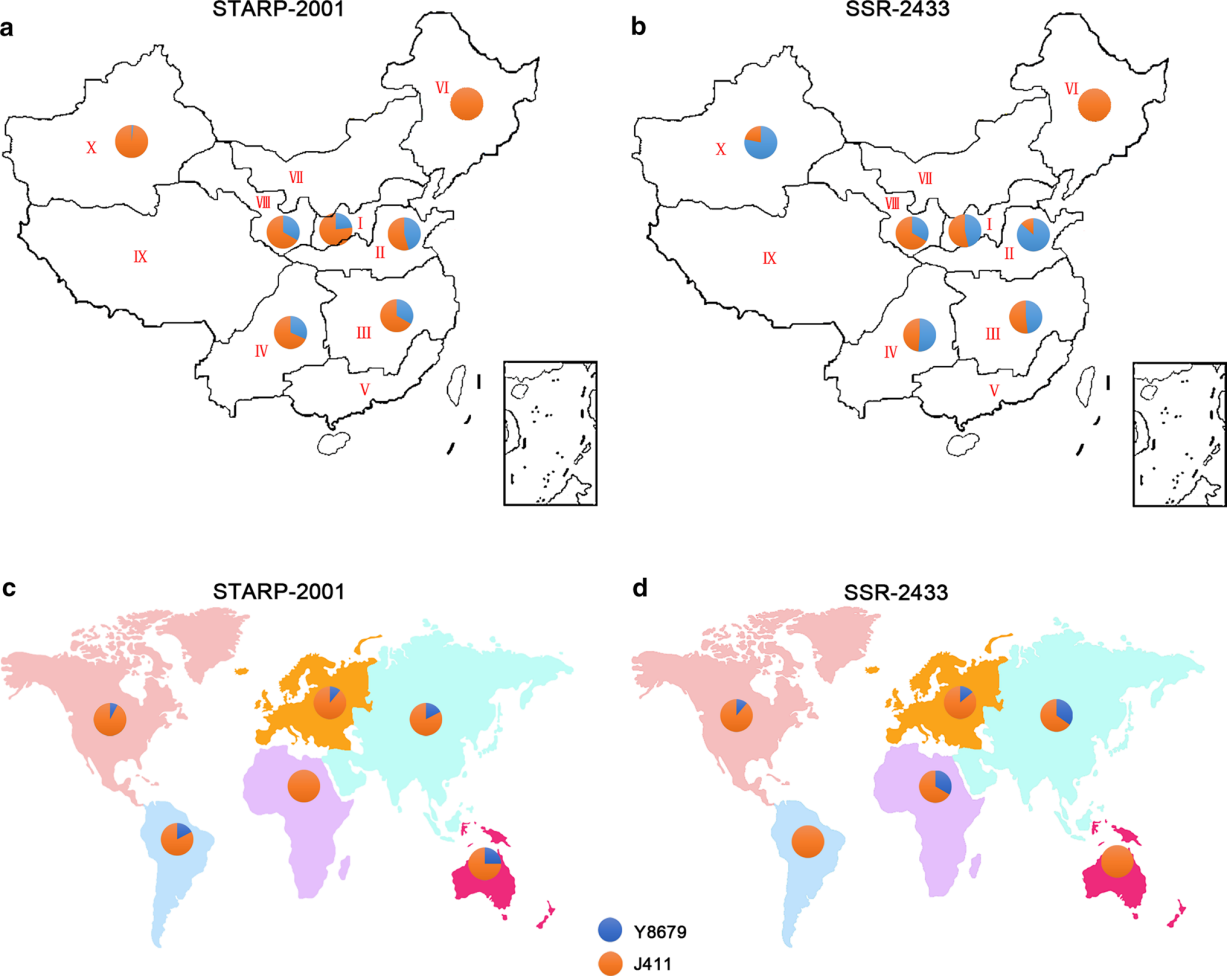
Haplotype distributions of *STARP*-*2001* and *SSR*-*2433* in 724 common wheat varieties/lines from China (**a**, **b**) and 442 common wheat varieties/lines from other countries (**c**, **d**). I, Northern winter wheat region; II, Yellow and Huai River valley winter wheat region; III, low and middle Yangtze River valley winter wheat region; IV, south-western winter wheat region; V, southern winter wheat region; VI, north-eastern spring wheat region; VII, northern spring wheat region; VIII, north-western spring wheat region; IX, Qinghai–Tibet spring–winter wheat region; X, Xinjiang winter–spring wheat region

### Effects of *QPht/Sl.cau*-*2D.1* and *QPht/Sl.cau*-*2D.2* on culm cell length

**Fig. 6:**
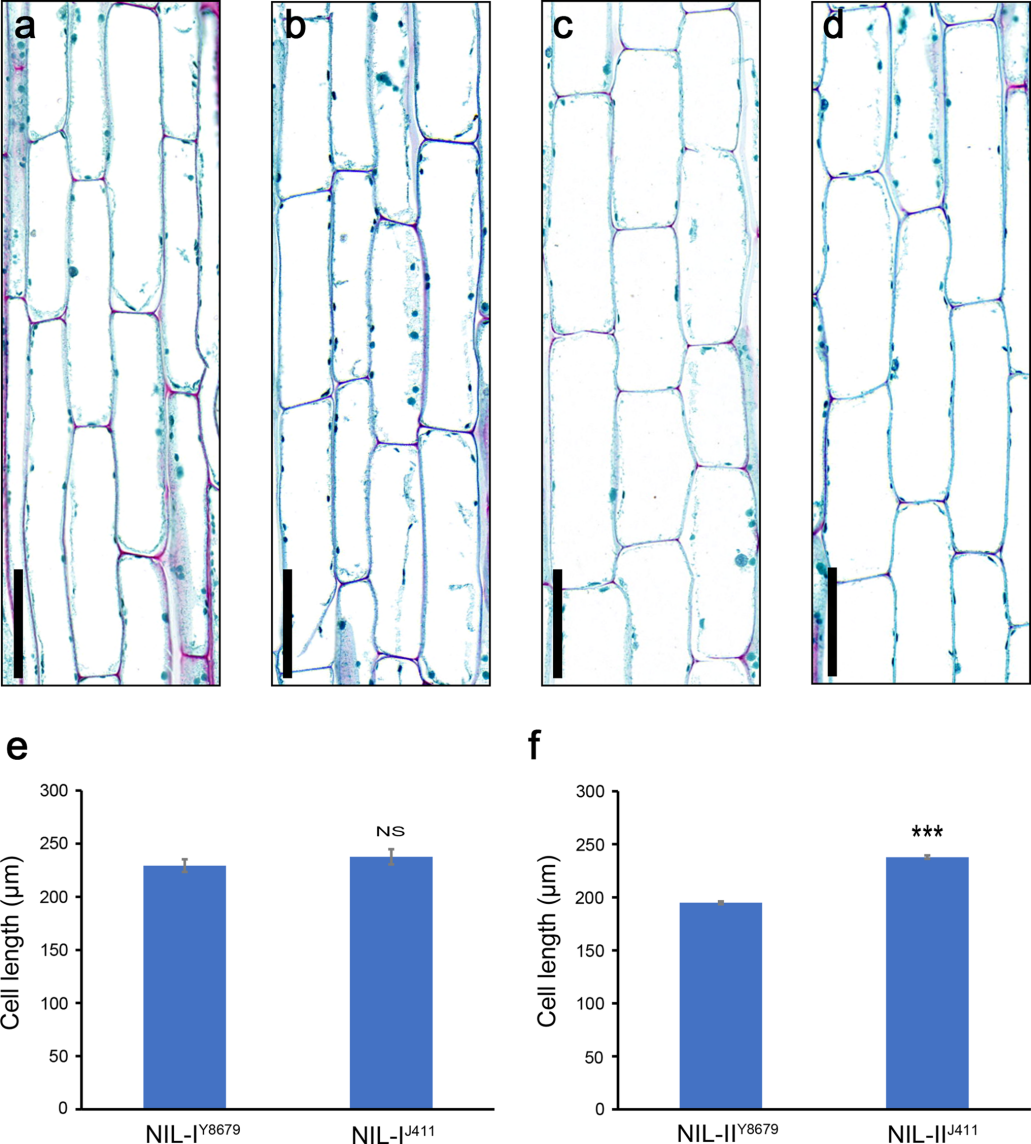
Longitudinal culm sections of NILs of *QPht/Sl.cau*-*2D.1* and *QPht/Sl.cau*-*2D.2* from the flowering stage. Scanning micrographs of the medial zone of the fully elongated peduncle in **a** NIL-I^Y8679^, **b** NIL-I^J411^, **c** NIL-II^Y8679^ and **d** NIL-II^J411^. Bars = 100 μm. Comparisons of parenchymatic cell length (μm) in medial sections of the peduncle from **e** NILs of *QPht/Sl.cau*-*2D.1* and **f***QPht/Sl.cau*-*2D.2*. NS, no significance *P* = 0.05; ***, *t* test *P *< 0.001. Bars represent the standard deviation

In wheat, internode elongation is caused by cell division and/or cell elongation (Chowdhry and Allan 1966; Gasperini et al. 2012). To investigate the underlying physiological bases of two QTL, longitudinal cell length in the median section of peduncle at the flowering stage in NILs of each QTL was measured. For *QPht/Sl.cau*-*2D.1*, NIL-I^Y8679^ showed no difference in cells length compared to NIL-I^J411^ (Fig. 6a, b and e). By contrast, there was significant difference in cell length between NIL-II^Y8679^ and NIL-II^J411^ of *QPht/Sl.cau*-*2D.2.* The cell length of NIL-II^J411^ with increasing allele was significantly longer than that of NIL-II^Y8679^ with decreasing allele for PHT (Fig. 6c, d, and f, *P *< 0.001).

### Haplotype analysis of *QPht/Sl.cau*-*2D.1* and *QPht/Sl.cau*-*2D.2*

**Fig. 7:**
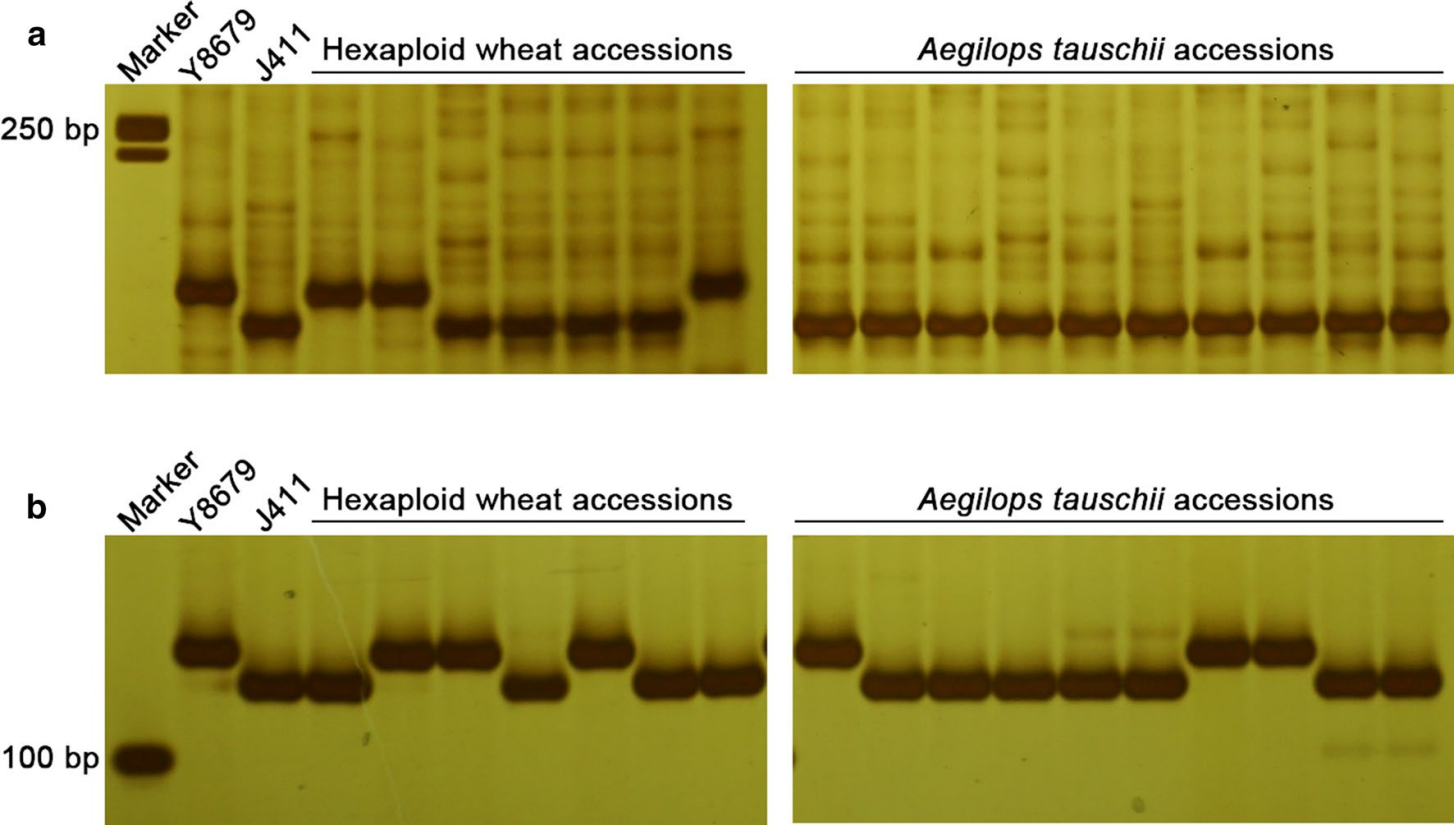
PCR products of *STARP*-*2001* (**a**) and *SSR*-*2433* (**b**) in several hexaploid wheat accessions and *Aegilops tauschii* accessions

To analyse the haplotype distribution of *QPht/Sl.cau*-*2D.1* and *QPht/Sl.cau*-*2D.2*, one SNP maker (AX-108988107) was converted into convenient STARP marker (*STARP*-*2001*) (Fig. 7a), which was used as the diagnostic marker of *QPht/Sl.cau*-*2D.1*. In addition, *SSR*-*2433* (Fig. 7b) was selected as the diagnostic marker for *QPht/Sl.cau*-*2D.2*. To test the efficiency of these two markers, the 191 RIL populations were genotyped and analysed. As expected, the group of STARP-2001-J allele had higher PHT and longer SL than that of STARP-2001-Y allele, with differences of 3.3–5.86 and 10.65–13.87% (*P *< 0.05) under different environments (Fig. 4a, b). Similarly, PHT and SL of the SSR-2433-J allele were much higher (*P *< 0.001) than those of the SSR-2433-Y allele (5.10–7.07 and 12.37–15.73%) (Fig. 4c, d). Collectively, our data exhibited that the alleles of STARP-2001-Y and SSR-2433-Y were significantly associated with the height-reduced alleles of *QPht/Sl.cau*-*2D.1* and *QPht/Sl.cau*-*2D.2,* respectively.

To further explore implications of our findings on wheat breeding, the haplotype of 724 common wheat varieties/lines from China was first analysed using diagnostic markers *STARP*-*2001* and *SSR*-*2433*. As shown in Fig. 5, both the STARP-2001-Y and SSR-2433-Y alleles were present in 6 of 10 agro-ecological production zones, including I, II, III, IV, VIII and X. The frequency of STARP-2001-Y was highest in zone II (45.60%), followed by zones III (33.33%), VIII (33.33%), IV (31.71%), I (23.26%) and X (1.83%) (Fig. 5a, Supplementary Table S11). The frequency of SSR-2433-Y allele in different zones was in the order of II (86.67%) > X (78.05%) > IV (51.22%) > III (48.15%) > I (46.51%) > VIII (33.33%) (Fig. 5b, Supplementary Table S11). When comparing the alleles of STARP-2001-Y and SSR-2433-Y in the same zone, the frequency of SSR-2433-Y was much higher than that of STARP-2001-Y in zones I, II, III, IV and X, especially in the major wheat production zone X (Fig. 5a, b, Supplementary Table S11). Furthermore, 442 common wheat varieties/lines from other countries were tested by the two diagnostic markers. The STARP-2001-Y allele was present in 48 of 442 accessions (10.86%), spreading in five continents, including Asia, Europe, North America, South America and Oceania. The SSR-2433-Y allele was detected in 63 of 442 accessions (14.25%), which are derived from Asia, Europe, Africa and North America (Fig. 5c, d, Supplementary Table S12).

To trace the origin of dwarf genes in the QTL intervals of *QPht/Sl.cau*-*2D.1* and *QPht/Sl.cau*-*2D.2,* mini core common wheat collections in China with 181 accessions were further genotyped using diagnostic markers *STARP*-*2001* and *SSR*-*2433*. The STARP-2001-Y allele was detected in 20 of 181 accessions (11.04%). By contrast, SSR-2433-Y allele presented in 73 of 181 accessions (40.33%) (Supplementary Table S2). In addition, a diverse panel of 86 accessions of *Aegilops tauschii*, the D-genome progenitor of *T. aestivum*, was also analysed. The results exhibited that the STARP-2001-Y allele was not detected in 86 accessions of *Aegilops tauschii*, but the SSR-2433-Y allele presented in 39 of 86 accessions (45.35%) (Supplementary Table S4).

### Sequence variations in candidate genes for *QPht/Sl.cau*-*2D.1* and *Rht8*

**Fig. 8:**
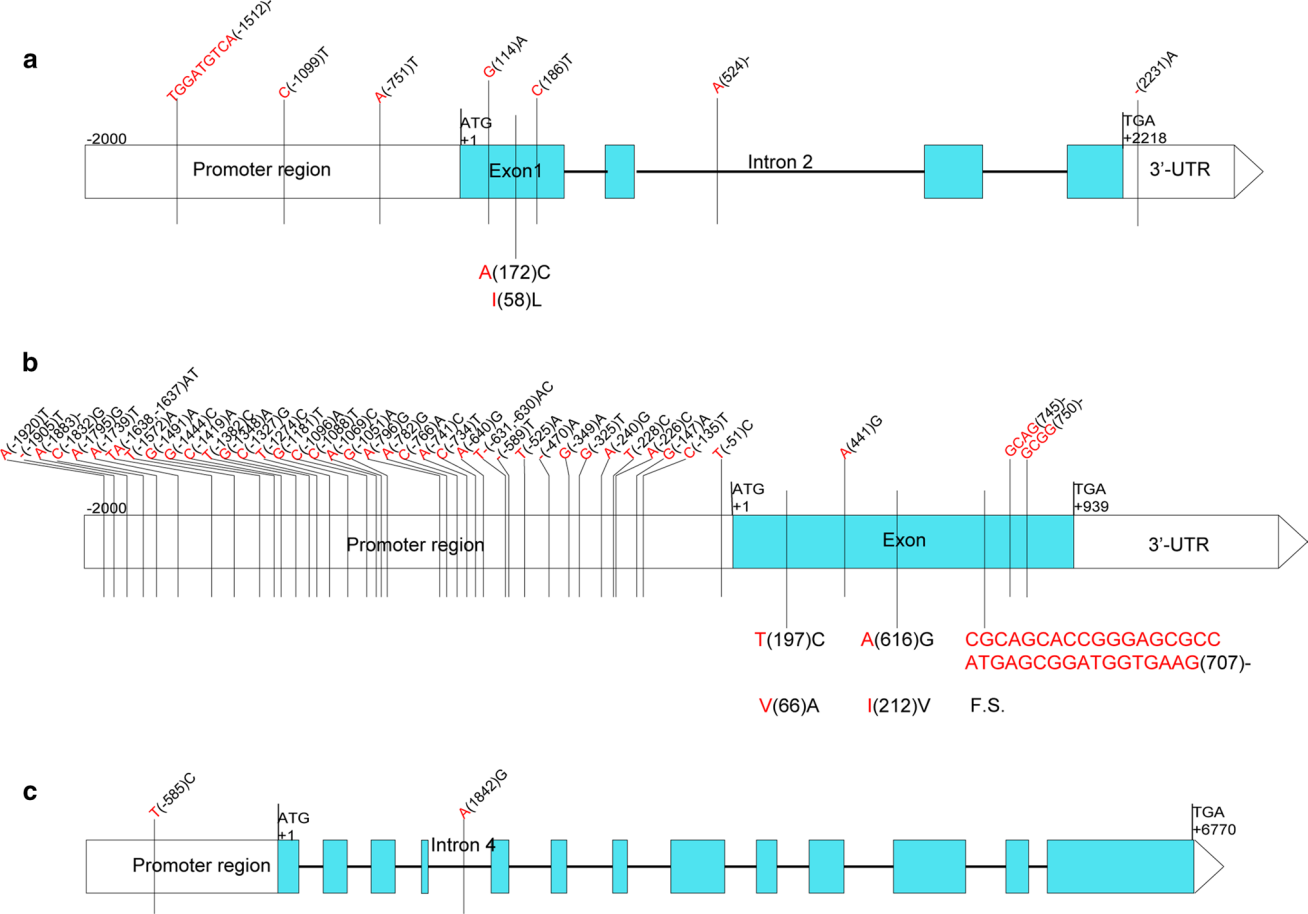
Structure of candidate genes showing the nucleotide and amino acid sequences polymorphism between Y8679 and J411. Lines, blue boxes and white boxes represent introns, exons and untranslated regions in the gene, respectively. Nucleotide and amino acid sequences of Y8679 and J411 are shown in red and black font, respectively. The numbers in bracket represent the positions of nucleotide or amino acid sequences relative to ATG. – represents deletion. F.S. indicates frame shift. **a** Structure of *TraesCS2D01G051500*. **b** Structure of *TraesCS2D01G055700*. **c** Structure of *TraesCS2D01G058700* (color figure online)

To identify the candidate genes for *QPht/Sl.cau*-*2D.1* and *Rht8*, we analysed the gene models in the mapping interval of the Chinese Spring RefSeq v.1.0 sequence. In rice, WRKY transcription factor *Dlf1* and gibberellin-inactivating 2-beta-dioxygenase gene played important roles in the regulation of PHT (Cai et al. 2014; Lo et al. 2008). Interestingly, of 38 predicted genes in the 2.5-Mb interval of *QPht/Sl.cau*-*2D.1* (*BS00022234_51*-*BobWhite_rep_c63957_1472*), *TraesCS2D01G051000* and *TraesCS2D01G051500* encoded two WRKY transcription factors, and *TraesCS2D01G049700* encoded gibberellin 2-beta-dioxygenase, which may be the candidate genes (Supplementary Table S10). DNA sequence analysis showed that there were 5 SNPs and 3 InDels between *TraesCS2D01G051500* of parental lines Y8679 and J411, including 3 SNPs in exon 1, 1 InDel in 3′-UTR region, 1 InDel in intron 2 and 2 SNPs and 1 InDel in the upstream region of translation start codon (Fig. 8, Supplementary Table S15). However, no sequence difference between Y8679 and J411 was detected for *TraesCS2D01G049700* and *TraesCS2D01G051000*.

The well-known reduced height genes *Rht*-*B1* and *Rht*-*D1* in wheat and several plant height related genes in rice (*DLT*, *SMOS1/SHB* and *SLR1*) encoded transcriptional factors belong to the GRAS family (De Vleesschauwer et al. 2016; Qiao et al. 2017; Tong et al. 2009). In rice, *OsCCC1* was involved in cell elongation by regulating ion (Cl^−^, K^+^, and Na^+^) homoeostasis to maintain cellular osmotic potential, which affected PHT in turn (Chen et al. 2016). Based on the new reference sequence of Chinese Spring (RefSeq v.1.0), the mapped region of *Rht8* between *SSR*-*2062* and *Xgwm484* contained 419 predicted genes, among which *TraesCS2D01G055700* and *TraesCS2D01G058700* encoded two GRAS transcription factors, and *TraesCS2D01G059300* encoded a cation–chloride cotransporter, which may be the candidate genes (Supplementary Table S10). DNA sequence analysis revealed that there were 38 SNPs and 8 InDels between *TraesCS2D01G055700* of Y8679 and J411, 3 SNPs and 3 InDels in the exon, and 35 SNPs and 5 InDels in the upstream region of translation start codon. For *TraesCS2D01G058700*, only 2 SNP variants were found, which were located in intron 4 and the upstream region of translation start codon, respectively (Fig. 8, Supplementary Table S15). By contrast, the sequence of *TraesCS2D01G095300* in Y8679 was identical to that in J411.

## Discussions

### Two QTL for PHT/SL linked in coupling phase on chromosomes 2DS

Neighbouring QTL that are linked in coupling phase are commonly observed in primary QTL analysis (Fan et al. 2017; Wu et al. 2015). To date, many studies have tried to dissect QTL in coupling phase using NILs or residual heterozygous lines and found that coupling QTL were partially attributed to tightly linked independent QTL (Chemayek et al. 2017; Shen et al. 2011). For example, Wu et al. dissected two tightly linked QTL for PHT (*qPH3*) and yield per plant (*qYD3*) by using two rice near isogenic populations (Wu et al. 2015). In wheat, tight repulsion linkage between *Sr36* and *Sr39* was revealed by genetic, cytogenetic and molecular analyses (Chemayek et al. 2017). Recently, using 191 RILs derived from Y8679 and J411, two QTL (*QPht/Sl.cau*-*2D.1* and *QPht/Sl.cau*-*2D.2*) controlling PHT and SL were mapped on the short arm of chromosome 2D. However, it is difficult to conclude whether *QPht/Sl.cau*-*2D.1* is a shadow or genuine QTL because it is only 7 cM away from *QPht/Sl.cau*-*2D.2* (Zhai et al. 2016). Here, we dissected these two QTL (*QPht/Sl.cau*-*2D.1* and *QPht/Sl.cau*-*2D.2*) using segregating populations and NILs derived from a residual heterozygous line (RIL171). They were linked in coupling phase on chromosome 2DS, with increased alleles from the same parent (J411). *QPht/Sl.cau*-*2D.1* was located between SNP markers *BS00022234_51* and *BobWhite_rep_c63957_1472*, and *QPht/Sl.cau*-*2D.2* was located between SSR markers *SSR*-*2062* and *Xgwm484*. The results of NILs indicated that the genetic effect of these two QTL is similar, which could reduce plant height by 4.79–14.32 and 9.49–14.43 cm under different environments (Table 2). Our present study provides a new example of QTL dissection in wheat, but the underlying molecular basis of two QTL (*QPht/Sl.cau*-*2D.1* and *QPht/Sl.cau*-*2D.2*) is still an area for further elucidation.

### Robust diagnostic SSR marker for *QPht/Sl.cau*-*2D.2*

Numerous studies exhibited that there were major QTL/genes on chromosome 2DS, such as the well-known and widely used dwarf gene *Rht8* (Chebotar et al. 2013; Griffiths et al. 2012; Zanke et al. 2014). Recently, using a fine-resolution mapping approach, the *Rht8* genetic interval has been reduced from 20.5 to 1.29 cM, flanking by *DG279* and *DG371* (Gasperini et al. 2012). Comparative analysis revealed that *QPht/Sl.cau*-*2D.2* of the present study is located on the same genetic interval of *Rht8* (Gasperini et al. 2012). Moreover, consistent with the effect of *Rht8* on culm cell elongation, significant difference in cell length was also observed between NILs of *QPht/Sl.cau*-*2D.2* (Fig. 6c, d). This, we speculated that *QPht/Sl.cau*-*2D.2* may be the well-characterized *Rht8* gene.

Due to the widespread use of *Rht8* in wheat breeding, assessing the distribution of height-reducing alleles at the *Rht8* is of interest for breeding purposes. Following the identification of *Xgwm261*, a closely linked marker to *Rht8* with the 192-bp allele corresponding to a height-reducing phenotype, the *gwm261*_192bp_ allele has been taken as diagnostic marker for *Rht8* (Ahmad and Sorrells 2002; Bai et al. 2004; Chebotar et al. 2001; Liu et al. 2005; Zhang et al. 2006). However, several instances were found in which the 192-bp allele was not associated with a height-reducing phenotype (Ellis et al. 2007). For example, a source variety of the Green Revolution semi-dwarfing genes, Norin10, carrying a 192-bp allele of *Xgwm261*, was independent of the height reduction effect of *Rht8* (Ellis et al. 2007). Consistent with this, our data revealed that *Xgwm261* was located in the genetic interval of *QPht/Sl.cau*-*2D.1,* instead of *QPht/Sl.cau*-*2D.2*. Thus, we developed a codominant SSR marker (*SSR*-*2433*) that was tightly linked to *QPht/Sl.cau*-*2D.2/Rht8*. Moreover, the *SSR*-*2433* locus only has two allelic variants in the diversity panel of wheat accessions, which may be an ideal diagnostic marker for the *QPht/Sl.cau*-*2D.2/Rht8* gene.

### A novel QTL for PHT/SL tightly linked to dwarf gene *Rht8*

Previous studies exhibited that numerous QTL/genes controlling PHT were detected in wheat (Borner et al. 2002; Gao et al. 2015a, b; Peng et al. 2003; Quarrie et al. 2005; Singh et al. 2016; Tian et al. 2017). Specifically, 24 dwarfing genes (*Rht1*–*Rht24*) have been catalogued in wheat (McIntosh et al. 2017). However, quite a few genes for reduced stature have been used in wheat breeding, because most showed strongly negative effects on grain yield (Chapman et al. 2007; Zhang et al. 2013a). Thus, it is necessary to explore and utilize new QTL/genes controlling PHT. For example, the new reduced PHT gene *Rht24* was important and extensively used in wheat breeding programmes (Wurschum et al. 2017). In the present and our previous studies, we identified a novel QTL for PHT neighbouring to *Rht8*, *QPht/Sl.cau*-*2D.1*, by the following evidences: (1) *QPht/Sl.cau*-*2D.1* was mapped to a position distal to *Rht8* on the short arm of chromosome 2D (Fig. 2a, Table 1); (2) *QPht/Sl.cau*-*2D.1* had no significant effect on culm cell elongation, which is obviously different from that of *Rht8* (Fig. 6).

Common wheat has an allohexaploid genome structure, which was hybrid by *T. turgidum* (AABB) and *Aegilops tauschii* (DD) 0.43 million years ago (International Wheat Genome Sequencing 2014; Petersen et al. 2006) To investigate the origin of the novel *QPht/Sl.cau*-*2D.1* during wheat evolution, we analysed the allelic variation in the diagnostic marker *STARP*-*2001* in a diverse panel of 86 accessions of *Aegilops tauschii.,* but the STARP-2001-Y allele was not detected, which is significantly associated with the height-reduced allele of *QPht/Sl.cau*-*2D.1*. Thus, it seems that the novel height-reduced allele of *QPht/Sl.cau*-*2D.1* was generated after the formation of allohexaploid wheat. Notably, haplotype analysis revealed that the height-reduced allele of *QPht/Sl.cau*-*2D.1* has not been widely used in wheat breeding compared to *Rht8.* Thus, *QPht/Sl.cau*-*2D.1* may be a favourable QTL in the genetic improvement in PHT in wheat, but the effect of different alleles of *QPht/Sl.cau*-*2D.1* on grain yield and their potential utilization needs further investigation.

### Candidate genes of *Rht8* and *QPht/Sl.cau*-*2D.1*

To date, several genes controlled PHT have been cloned in wheat, such as *Rht*-*B1*, *Rht*-*D1, TaSTE* and *GA2oxA9* (*Rht18*) (Ford et al. 2018; Gasperini et al. 2012; Peng et al. 1999; Zhang et al. 2013b). Specifically, *Rht*-*B1* and *Rht*-*D1* encoded transcriptional factors belong to the GRAS family (Peng et al. 1999; Sun 2010). Different alleles of *Rht*-*B1* and *Rht*-*D1* have been also identified, which produce dwarfs with a broad range of plant height (Pearce et al. 2011). Semi-dwarfing alleles *Rht*-*B1d* and *Rht*-*B1e* introduce premature stop codons within the amino-terminal coding region, whereas the severe dwarfism of *Rht*-*B1c* gene is caused by an intragenic insertion of 90 bp within the highly conserved amino-terminal DELLA domain (Pearce et al. 2011). In the present study, we found that two of the three candidate genes for *Rht8* (*TraesCS2D01G055700* and *TraesCS2D01G058700*) encoded GRAS transcription factors. Notably, a 35-bp deletion in the coding region of *TraesCS2D01G055700* was found in parental line J411 as compared to Y8679, which resulted in frame shift for translation. Moreover, another two SNP variations between Y8679 and J411 leaded to amino acid substitutions. In addition, 35 SNPs and 5 InDels in the upstream region of translation start codon were detected (Fig. 8, Supplementary Table S15). Collectively, we proposed that the role of *TraesCS2D01G055700* in plant height merit for further investigation.

In rice, *Dlf1*, a WRKY transcription factor, acts as a transactivator to downregulate *Ehd2/RID1/OsId1* in the signal transduction pathway of flowering and plays an important role in the regulation of PHT (Cai et al. 2014). Of the three candidate genes for *QPht/Sl.cau*-*2D.1, TraesCS2D01G051000* and *TraesCS2D01G051500* encoded two WRKY transcription factors, but only *TraesCS2D01G051500* has sequence variations between Y8679 and J411, including one SNP (A/C) in exon 1 that leaded to the substitution of 1 amino acid (*I*/*L*) and 2 SNPs and 1 InDel in the upstream region of translation start codon (Fig. 8, Supplementary Table S15). Therefore, detailed studies would be necessary to analyse the effect of these variations in the function of *TraesCS2D01G051500* and plant height.

Epigenetics regulate gene expression without effects on gene sequences, which was involved in diverse aspects of plant development (Bender 2002; Heer et al. 2018; Paszkowski and Mittelsten Scheid 1998). The repression of *FLC* expression in Arabidopsis by vernalization is one of the well-studied examples of the epigenetic regulation of a gene in plants (Bastow et al. 2004; Questa et al. 2016). Recently, Zhang et al.(2012) identify a DNA hypomethylation epi-allele in rice and demonstrate that repression of *FIE1* via DNA methylation and H3K9me2 is essential for plant height. Thereby, although the sequences of *TraesCS2D01G049700*, *TraesCS2D01G051000* and *TraesCS2D01G095300* in Y8679 were identical to those in J411, these genes may also be the candidate genes and further experiment is needed to validate which one is the real gene for *Rht8* and *QPht/Sl.cau*-*2D.1*.

In conclusion, two QTL with pleiotropic effects on plant height and spike length linked in coupling phase on chromosome 2DS in common wheat were separated, and the diagnostic marker tightly linked with each QTL was developed, which corresponded to a novel QTL *QPht/Sl.cau*-*2D.1* and well-known *Rht8* gene, respectively. Of six candidate genes for two QTL, three have sequence variations between parental lines Y8679 and J411. Remarkably, the frequency of the height-reduced allele of *QPht/Sl.cau*-*2D.1* in diverse panels of wheat accessions is much lower than that of *QPht/Sl.cau*-*2D.2*. Taken together, these data provide a new example of QTL dissection, and *QPht/Sl.cau*-*2D.1* may be an attractive target for genetic improvement in wheat breeding.

#### Author contribution statement

ZN and AZ conceived the project; LC, ZC and RB carried out experiments; XC and HZ participated in field trials; LC, ZC and XC performed marker development of the QTL region of interest; LC and ZH developed the segregation populations and near isogenic lines; LC, ZC and RB performed phenotyping of the segregation populations and the near isogenic lines; QS, HP, YY, ZH, MX and WG assisted in revising the manuscript; LC analysed experimental results and wrote the manuscript.

## Electronic supplementary material

Below is the link to the electronic supplementary material.

Supplementary material 1 (XLSX 187 kb)
